# Neuroprotective Activities of *Crossyne flava* Bulbs and Amaryllidaceae Alkaloids: Implications for Parkinson’s Disease

**DOI:** 10.3390/molecules26133990

**Published:** 2021-06-30

**Authors:** Sylvester I. Omoruyi, Abobaker S. Ibrakaw, Okobi E. Ekpo, James S. Boatwright, Christopher N. Cupido, Ahmed A. Hussein

**Affiliations:** 1 Department of Chemistry, Cape Peninsula University of Technology, Symphony Road, Bellville 7535, South Africa; omoruyis@cput.ac.za; 2 Department of Biodiversity and Conservation Biology, University of the Western Cape, Cape Town, Robert Sobukwe Road, Bellville 7535, South Africa; 3686844@myuwc.ac.za (A.S.I.); jboatwright@uwc.ac.za (J.S.B.); 3 Department of Anatomy and Cellular Biology, College of Medicine and Health Sciences, Khalifa University, Abu Dhabi P.O. Box 127788, United Arab Emirates; okobi.ekpo@ku.ac.ae; 4 Department of Botany, University of Fort Hare, Private Bag X1314, Alice 5700, South Africa; CCupido@ufh.ac.za

**Keywords:** Amaryllidaceae, alkaloids, *Crossyne flava*, Parkinson’s disease, 1-methyl-4-phenylpyridinium (MPP^+^), neuroprotection

## Abstract

Parkinson’s disease (PD) is one of the most common neurodegenerative diseases and affects approximately 6.3 million people worldwide. To date, the treatment of PD remains a challenge, as available treatment options are known to be associated with serious side effects; hence, the search for new treatment strategies is critical. Extracts from the Amaryllidaceae plant family as well as their alkaloids have been reported to have neuroprotective potentials. This study, therefore, investigated the biological activities of *Crossyne flava* and its isolated alkaloids in an in vitro MPP^+^ (1-methyl-4-phenylpyridinium) PD model using SH-SY5Y cells. The effects of the total extract as well as the four compounds isolated from *Crossyne flava* (i.e., pancratinine B (**1**), bufanidrine (**2**), buphanisine (**3**), and epibuphanisine (**4**)) were evaluated for cell viability, neuroprotection, levels of reactive oxygen species (ROS), adenosine triphosphate activity (ATP), and caspase 3/7 activity in SH-SY5Y cells. The results obtained showed that pre-treatment with both the extract and the isolated compounds was effective in protecting the SH-SY5Y cells from MPP^+^-induced neurotoxicity and inhibited ROS generation, ATP depletion as well as apoptosis induction in the SH-SY5Y cells. The results of this study show that the Amaryllidaceae plant family may be a source of novel compounds for the treatment of neurodegenerative diseases, which validates the reported traditional uses.

## 1. Introduction

Parkinson’s disease (PD) is the second most common neurodegenerative disease, affecting approximately 6.3 million people worldwide, and it is characterized by dopaminergic neuronal loss in the substantia nigra pars compacta part of the mid-brain [1]. Parkinsonism is a motor standpoint for the clinical diagnosis of PD, as it encompasses four PD symptoms including resting tremor, rigidity, bradykinesia, and postural instability [2]. Other than the dopaminergic loss, PD is pathologically marked by the presence of intraneuronal proteinaceous cytoplasmic inclusions made of alpha-synuclein proteins that accumulate in Lewy neurites and Lewy bodies [3,4]. Inadequate dopamine (a very vital brain monoamine known to function primarily as an inhibitory neurotransmitter) levels, make it hard to regulate the striatal neurons excitability leading to degeneration and making the ability to control movements very difficult for patients [5].

Evolutionary advances in the past two decades have supported findings that understanding PD progression is not only compromised by genetic factors but also by the association of environmental toxins with free radical formation and oxidative stress [6]. These environmental factors include tobacco use and chemical/pesticide exposure [7,8], a notable chemical being MPTP (1-methyl-4-phenyl-1,2,3,6-tetrahydropyridine), which is a pro-drug to the neurotoxin MPP^+^ used in this study. MPP^+^ is a metabolite of MPTP and causes permanent PD symptoms, especially the death of dopaminergic neurons. MPP^+^ exerts its neurotoxic activities through the generation of free radicals, which induces the inhibition of the mitochondrial complex I electron transport chain and, in turn, causes a depletion of adenosine triphosphate (ATP) that leads to neuronal cell death.

Presently, there have been no successful disease-modifying treatments to halt the progression of PD, hence, current and upcoming research has been focused on discovering more therapeutic approaches for alleviating the motor symptoms of PD [9]. These treatment approaches are often administered based on the severity of the symptoms and side effects experienced by individual patients [10]. The most common form of treatment is the use of levodopa which has been shown to also lead to PD-like symptoms over time. This challenge has given room to more investigations into the search for novel therapies which include both synthetic and plant-derived natural products that could be used as effective alternative treatments for PD [11].

For hundreds of years, medicinal plants have been used in many healthcare systems in different parts of the world as safe, efficacious, acceptable treatments for a wide variety of disease conditions with little or no side effects reported compared to chemical-based drugs [12]. Some discoveries in the past decade have increased the reliance on medicinal plants in both developing and developed countries, which has led to a remarkable surge in the acceptance of herbal remedies as a primary source of healthcare and medicinal products [13]. Herbal medicines have been used by specialists as adjuvant treatment for PD, to lessen the dosage of dopaminergic drugs and to improve the side effects that come with prolonged usage of these drugs [14,15]. A notable plant family with diverse biological activities is the Amaryllidaceae family and its species have been used traditionally for nervous system-related conditions [16].

Amaryllidaceae are largely domiciled in the tropical and temperate regions of the world and consist of over 800 species and about 60 genera [17]. Plants from this family are known for their alkaloids, which have been reported to have several beneficial biological activities including antiviral, antibacterial, antifungal, antimalarial, analgesic anti-cancer, and neuroprotective activities [18,19,20,21]. Furthermore, the Amaryllidaceae alkaloid galanthamine received approval from the United States Food and Drug Administration (FDA) for the treatment of Alzheimer’s disease (AD) [22]; hence, the exploitation of Amaryllidaceae alkaloids for novel bioactive compounds with neuroprotective activity is plausible [20,21].

The deciduous bulb plant, *Crossyne flava* (*C. flava*), is a member of the Amaryllidaceae family, which can grow up to 50 cm in height, ranging in size from 9 to 13 cm. It is majorly distributed in the West Coast of South Africa, has been studied previously under the name of *Boophone flava*, and is known to contain 14 alkaloids (Viladomat et al., 1995a). To the best of our knowledge, there is no reported study on the neuroprotective activities of *C. flava*. The present study therefore investigated the neuroprotective activities of *C. flava* and its bioactive compounds on MPP^+^-induced neurotoxicity in SH-SY5Y cells.

## 2. Results

### 2.1. Identification of Compounds

**Figure 1:**
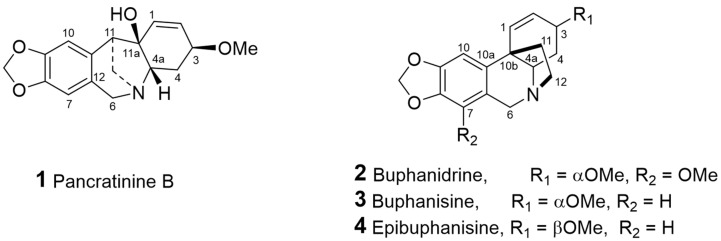
Chemical structures of isolated compounds **1**–**4** from *C. flava*.

Four known alkaloids (**1**–**4**; Figure 1) were isolated and identified based on NMR and GC-MS analysis (Table 1). Compounds **2**–**4** were identified as bufanidrine (**2**), buphanisine (**3**), and epibuphanisine (**4**) [23,24]. These compounds belong to crinine-type alkaloids, and according to the recent review by Berkov et al., so far there are 85 compounds isolated from the Amaryllidaceae family and with a crinine skeleton [25]. The first compound (i.e., pancratinine B) belongs to montanine-type alkaloids and was isolated once from Pancratium canariense [26].

### 2.2. Dose Response of C. flava and Compounds

**Figure 2:**
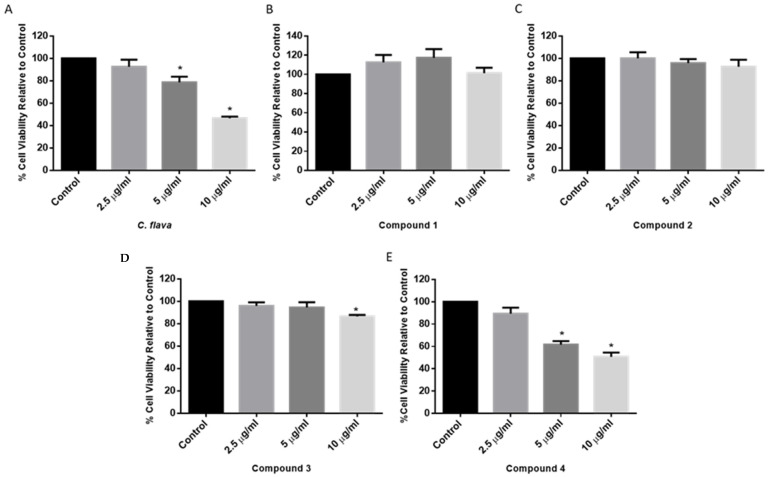
Dose–response of *C. flava* and compounds. MTT assay cytotoxicity on SH-SY5Y cells treated with increasing concentrations (2.5, 5, and 10 µg/mL) of *C. flava* (**A**) compounds **1**, **2**, **3**, and **4** (**B**–**E**) for 24 h. Each bar represents mean cell viability expressed as a percentage of the control. * Indicates significance at *p* < 0.05.

To determine the optimal dose of *C. flava* and compounds that will show neuroprotection, a cell viability assay was performed in the SH-SY5Y cells treated with 2.5, 5, and 10 µg/mL of either *C. flava* extract or compounds. The results show that the total extract induced a dose-dependent reduction in cell viability, which was only significant at the 5 and 10 µg/mL concentrations (Figure 2A). For the compound-treated cells, compound **1** showed a moderate increase in cell viability at concentrations tested albeit not significant, while compound **2** showed a slightly concentration-dependent reduction in cell viability, which was also not significant (Figure 2B,C). In addition, compounds **3** and **4** also showed a concentration-dependent reduction in cell viability and was significant at 10 µg/mL for **3** and at both 5 and 10 µg/mL for **4** when compared to the control (cells treated with a similar concentration of DMSO in the highest concentration of the compounds) (Figure 2D,E). Together, the *C. flava* extract did not induce any marked changes in the cell viability of SH-SY5Y cells at the 2.5 µg/mL concentration, and this was similar for the compounds. Hence, this was selected as the optimum concentration to be used for further neuroprotection experiments.

### 2.3. C. flava and Compounds Mitigated MPP^+^-Induced Toxicity

**Figure 3:**
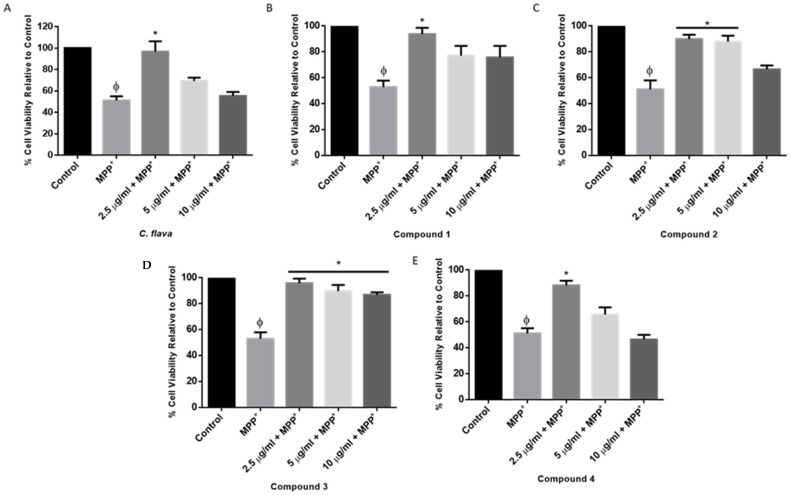
*Crossyne flava* and compounds showed protection in SH-SY5Y cells. Cells were pre-treated with *C. flava* (2.5, 5, and 10 µg/mL) (**A**) and compounds **1**, **2**, **3**, and **4** (**B**–**E**) before exposure to MPP^+^ for 24 h. Each bar represents the mean percentage cell viability relative to the control, and the significance of the difference is indicated by * *p* < 0.05 when extract/compounds are compared to MPP^+^-treated cells. ϕ Represents MPP^+^ vs. control.

To determine whether *C. flava* or compounds protect SH-SY5Y cells from the MPP^+^-induced toxicity, 10,000 cells were plated per well and treated with 2.5 µg/mL of either extract or compounds for 2 h before the addition of 2000 µM MPP^+^ and, thereafter, an MTT assay was performed after 24 h. The results show that treatment of SH-SY5Y cells with MPP^+^ led to a significant reduction in SH-SY5Y cell viability when compared to the control. Indeed MPP^+^ reduced cell viability to approximately 40–50%, while in the cells that were exposed to *C. flava* prior to the addition of MPP^+^, cell viability was seen to be improving towards normal, and this was dose dependent. Indeed, cell viability was 96.5%, 69.3%, and 55.3%, respectively, for the 2.5, 5, and 10 µg/mL concentrations of the extract (Figure 3A). Similarly, all compounds showed neuroprotective activities, and this was significant, especially at the 2.5 µg/mL concentration (Figure 3B–E).

Interestingly, compound **3** showed significant neuroprotection at concentrations tested, while compound **2** was only significant at the 2 and 5 µg/mL concentrations. Together, *C. flava* and compounds conferred neuroprotection in SH-SY5Y cells exposed to MPP^+^ toxicity, and the 2.5 µg/mL showed the best activity and was selected as the optimum concentration to be used for further studies.

### 2.4. C. flava Prevented MPP^+^-Induced Alterations in Cell Morphology

**Figure 4:**
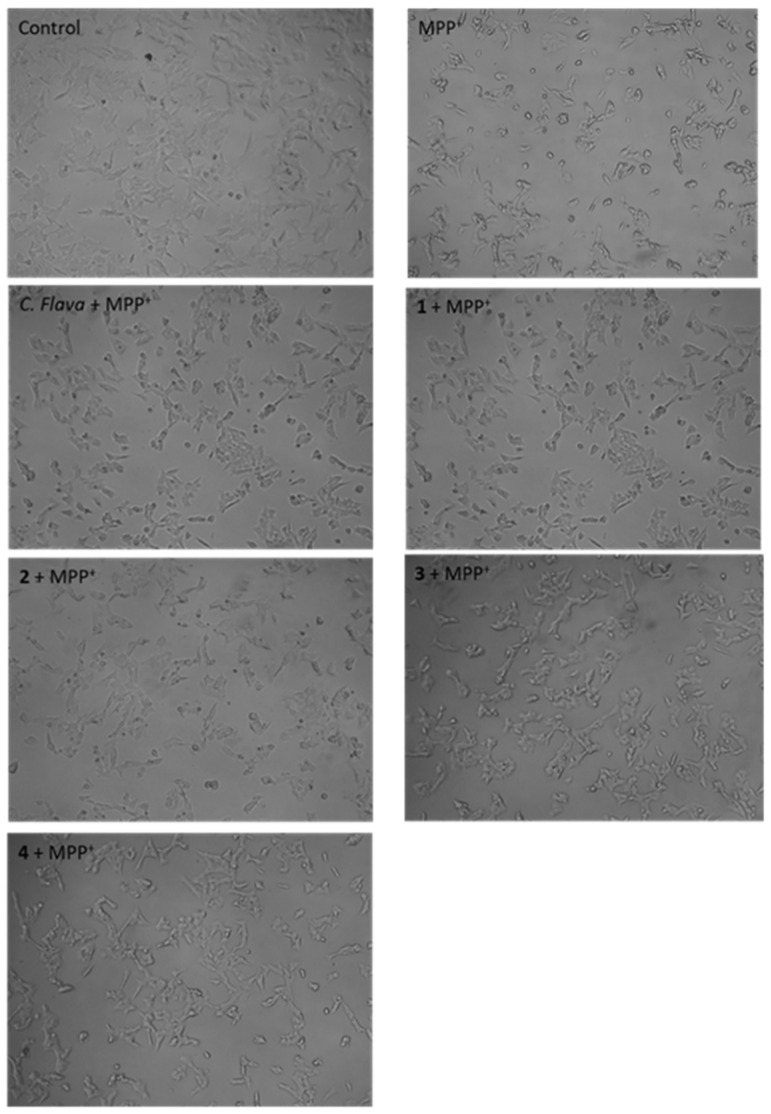
*Crossyne flava* inhibited SH-SY5Y morphological changes induced by MPP^+^. SH-SY5Y cells were pre-treated with total extract and compounds at the 2.5 µg/mL before exposure to 2000 µM MPP^+^ for 24 h. Cells were visualized, and images were acquired using the light microscope at 100× magnification.

Following the neuroprotection experiments, changes in the morphology of MPP^+^-alone exposed cells, as well as cells treated with the extract and compounds post-MPP^+^ exposure, were evaluated. The results showed, as expected, that MPP^+^ induced morphological changes in cells ranging from loss of neuronal projections, cell shrinkage to roundness of cells. However, cells pre-treated with *C. flava* and compounds showed improved cell morphology with minor changes when compared to the control (Figure 4). These results tend to indicate a restoration of cell morphology that may suggest a neuroprotection effect for *C. flava*.

### 2.5. C. flava and Compounds Mitigate MPP^+^-Induced ROS Generation

**Figure 5:**
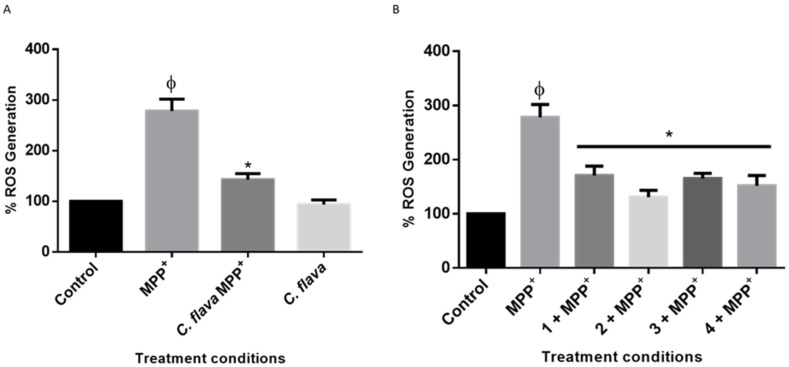
*Crossyne flava* and compounds mitigated ROS production induced by MPP^+^. SH-SY5Y cells were pre-treated with 2.5 µg/mL of *C. flava* (**A**) and 2.5 µg/mL of compounds (**B**) before exposure to MPP^+^ for 24 h, and ROS production was measured using DCFDA fluorescent dye. Each bar represents the mean fluorescent intensity expressed as a percentage of the control. The significance of the difference is indicated by * *p* < 0.05 when extract/compounds are compared to MPP^+^-treated cells. ϕ Represents MPP^+^ vs. the control.

To understand the mechanisms of action of *C. flava* and the compounds in MPP^+^-induced neurotoxicity in SH-SY5Y cells, the effects of the extract and compounds on ROS generation was next investigated. It is widely established that MPP^+^ treatment triggers ROS production; hence, the ability of neuroprotective agents to inhibit ROS production is a possible neuroprotection mechanism [27,28]. In this study, cells pre-treated with 2.5 µg/mL of the extract and the compounds before the addition of MPP^+^ significantly inhibited MPP^+^-induced ROS generation as seen in Figure 5. Taken together these results show that *C. flava* and the compounds mitigated ROS production induced by the neurotoxin, MPP^+^, in SH-SY5Y cells.

### 2.6. C. flava and Compounds Attenuated MPP^+^-Induced Loss of ATP

**Figure 6:**
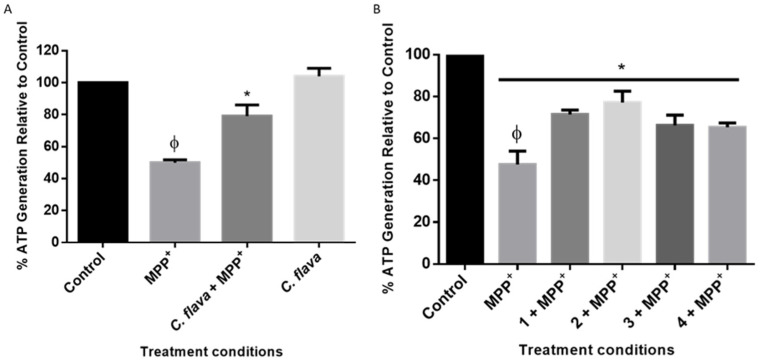
*Crossyne flava* and compounds attenuated MPP^+^-induced ATP degeneration. Cells were pre-treated with 2.5 µg/mL of *C. flava* (**A**) and compounds (**B**) before exposure to 2000 µM of MPP^+^ for 24 h and ATP levels assessed. Each bar represents the mean percentage level relative to the control, and the significance of the difference is indicated with * when extract/compounds are compared to MPP^+^ and ϕ (MPP^+^ vs. control).

As a pathology of PD, ATP is depleted following the generation of ROS [29,30]. Considering this, we next investigated the changes in ATP levels to ascertain if *C. flava* extract and compounds would inhibit the depletion of ATP in the SH-SY5Y cells. The results obtained showed that MPP^+^ treatment significantly reduced ATP levels in the cells when compared to the control, but pre-treatment with the extract and the compounds attenuated ATP levels in the cells for all treatment conditions (Figure 6).

### 2.7. C. flava and Compounds Inhibited MPP^+^-Induced Apoptosis

**Figure 7:**
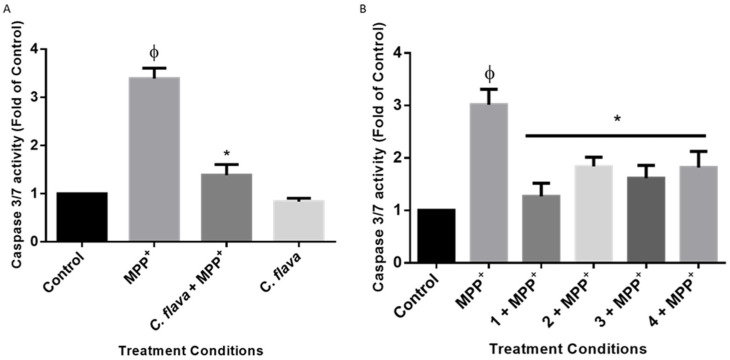
*C. flava* and compounds inhibited MPP^+^-induced caspase 3/7 activity. Cells were pre-treated with 2.5 µg/mL of extract (**A**) and compounds (**B**) before exposure to 2000 µM of MPP+ for 24 h and activity of caspase 3/7 was determined. Each bar represents the level of caspase 3/7 expressed as a fold of the control, and the significance of the difference is indicated with * when extract/compounds are compared to MPP^+^ and ϕ (MPP^+^ vs. control).

The reduction of ATP levels in PD is known to lead to cell death [31]. Thus, to further investigate the mechanism of neuroprotection by *C. flava* and the compounds, cells were treated prepared for the neuroprotection experiments as previously described, and the activities of caspase 3/7 was determined as an indicator for apoptosis. Caspases belong to a family of cysteine proteases that are known to be elevated during apoptosis, with their activities often linked to cell death [32]. Caspases could be initiators of apoptosis; caspases 8 and 9 are activated during the extrinsic and intrinsic apoptotic pathways, respectively, and the executioner caspases (i.e., 3 and 7) lie downstream of the apoptotic pathway [33]. The executioner caspases are often used to determine apoptosis when the aim is not to distinguish between the intrinsic and extrinsic apoptotic pathways [34,35]. The results of this study show that following MPP^+^ treatment alone, elevated levels of caspase 3/7 activities were evident. However, pre-treatment with *C. flava* and compounds mitigated the increase in the levels of caspase 3/7 towards the normal control (Figure 7). Together, these results show that the inhibition of apoptosis is one of the mechanisms of neuroprotection conferred by *C. flava* and its bioactive compounds.

## 3. Discussion

Parkinson’s’ disease continues to pose a challenge to quality of life, and despite the many years that have passed since this condition was first reported, treatment options are still lacking [36,37]. The most widely used treatment for PD is levodopa, which addresses only some of the symptoms and may lead to serious side effects if used for too long [38,39,40]. The present study investigated the neuroprotective effects of *C. flava* and its bioactive alkaloids in an in vitro PD model. We provide the first evidence of the neuroprotective activities of bioactive compounds derived from *C. flava* in a PD model and these compounds included pancratinine B (**1**), bufanidrine (**2**), buphanisine (**3**), and epibuphanisine. The findings show that both *C. flava* and its compounds attenuated the neurotoxicity induced by MPP^+^. These findings are consistent with the traditional uses of the Amaryllidaceae plant family, as a previous study reported that plants from this family were being used traditionally for the treatment of neurological disorders [41]. Some biological and pharmacological studies have also validated the claim of previous traditional uses [42,43,44,45].

Furthermore, the Amaryllidaceae plant family also has a unique family of alkaloids that have been shown to offer neuroprotective effects [46,47,48]. In this study, the four alkaloids isolated and identified demonstrated neuroprotective activities in the PD model. Interestingly, bufanidrine (**2**) and buphanisine (**3**) have previously been shown to have a strong affinity for the serotonin reuptake transport protein (SERT), suspected to be linked to the presence of the 1,3-dioxole moiety in the Amaryllidaceae alkaloids, which was considered to be responsible for their neuroprotective effects in AD [49,50,51]. Whether this moiety is also responsible for the anti-PD effects seen in this study is not yet known, but there is similarity in the pathways of the progression of both PD and AD which may be linked to oxidative stress and mitochondrial dysfunction [29,52,53]. Similarly, epibuphanisine (**4**) has also been shown to bind to SERT and the GABA_A_-benzodiazepine receptor and to inhibit acetylcholinesterase as a mechanism of action within the central nervous system, which can also be linked to anti-anxiety and anti-Alzheimer’s effects [50,54].

Mitochondrial oxidative stress is a mechanism in the pathology of PD in which there is impairment of the mitochondrial complex I and, subsequently, a depletion in ATP levels in brain cells [55]. Following MPP^+^ toxicity, a decrease in ATP as well as an increase in the levels of ROS production were observed that, in turn, mitigate other mitochondrial complexes, such as III and IV, as well as suppress mitochondrial function including mitochondrial gene expression, protein expression, and oxidative phosphorylation proteins [56,57,58]. In line with this, the findings from this study showed that *C. flava* and compounds prevented the accumulation of ROS in the cells following exposure to MPP^+^. In addition, dopamine and calcium signaling in the cells was also affected by ATP loss, all of which lead to PD [59]. Thus, the level of ATP generation in the cells is a critical test for mitochondrial integrity. The findings from the current study showed that *C. flava* and compounds attenuated MPP^+^-induced ATP depletion in cells, which provides an indication of improved mitochondrial function and integrity. Consistent with our findings, *Boophone disticha* belonging to the Amaryllidaceae family has been shown to repeal the effect of 6-hydroxydopamine (6-OHDA)-induced ATP loss in SH-SY5Y cells [60].

Additionally, ATP depletion in cells following neurotoxicity induced by MPP^+^ leads to cell death (either in the form of apoptosis or necrosis) [59,61]. Apoptosis is a form of programmed cell death that involves a cascade of events that either go via the intrinsic (or mitochondrial) pathway or the extrinsic pathway [33]. However, critical to the downstream of both pathways are the executioner caspases, such as caspase 3/7, which are effector caspases that promote cleavage of cellular content and eventual cell death [62]. The inhibition of apoptosis in neurodegenerative diseases has been suggested to be a therapeutic option [63], which is critical for the normal functioning of cells in PD progression. Findings from the current study showed that *C. flava* and compounds were able to rescue cells from apoptosis induced by MPP^+^ treatment. Importantly, it is difficult to infer from the current study whether the necrosis pathway was also activated, which will be worth investigating in future studies.

## 4. Materials and Methods

### 4.1. Chemical and Reagents

Organic solvents, such as methanol (HPLC grade), ethanol, ethyl acetate, and hexane, were supplied by Merck (Cape Town, South Africa). Thin layer chromatography (TLC) was performed on normal-phase (Merck) silica gel 60 PF_254_ pre-coated aluminum plates. Column chromatography was conducted on silica gel 60 H (0.040–0.063 mm particle size, Merck, Cape Town, South Africa) and Sephadex LH-20 (Sigma–Aldrich, Cape Town, South Africa).

The NMR spectra were recorded on an Avance 400 MHz NMR spectrometer (Bruker, Rheinstetten, Germany) in deuterated chloroform, using the solvent signals as the internal reference. The GC-MS analysis was performed utilizing an Agilent Technologies 7820A coupled with an MSD5977E. Samples of 1.0 mg were dissolved in 1.0 mL of CH_2_Cl_2_, and 1.0 μL was injected directly into the GC-MS operating in the electron ionization (EI) mode at 70 Ev and utilizing an HP5 MS column (30 m, 0.25 mm i.d., film thickness 0.25 m). The temperature gradient performed was adjusted as 40–80 °C (8 min), 80–220 °C (10 °C/min), hold at 220 °C for 5 min, 220–300 °C (20 °C/min), and 10 min hold at 300 °C. The injector and detector temperatures were both at 250 °C, with source and MS Quad at 230 °C and 150 °C, respectively, and the flowrate of the carrier gas (He) was 1.5 mL/min. A split ratio of 1:3 was applied.

### 4.2. Collection and Identification of the Plant Material

Bulbs of *C. flava* were collected in November 2018 in the Karoo National Gardens, Worcester, South Africa. The identity of the species was authenticated by one of the co-authors (CNC) and voucher specimens (UFH 20 April 2020) were deposited in the Giffen Herbarium of the University of Fort Hare (UFH), South Africa.

### 4.3. Preparation of Plant Extract and Isolation of Compounds

Fresh bulbs (150 g) of *C. flava* were blended and extracted with methanol for 2 days. The total extracts were combined and evaporated under reduced pressure at 40 °C to give a yield of 21 g. For isolation of compounds, 20 g of the total extract was loaded on a silica gel column (5 × 35 cm) and eluted with a gradient mixture of hexane and ethyl acetate of increasing polarity. Similar fractions were pooled together according to their TLC profile to give 25 main fractions. Fraction XI (63.7 mg) was chromatographed on Sephadex using isocratic 5% aqueous ethanol, then Prep-TLC to yield compound 1 (5.2 mg), while Fraction XV (108.2 mg) was chromatographed under the same conditions to yield compound 166.6 (7.2 mg). In addition, Fraction XIV (100.3 mg) was chromatographed on a silica gel column (using an isocratic elution of 5% DCM/MeOH) to yield compounds labeled as (170-1, 7.3 mg) and (170-2, 5.3 mg), respectively.

Pancratinine B (**1**) GC-MS: R_t_ 22.478 min; MS; *m*/*z*: 301.3 (C_17_H_19_NO_4_), 286.2, 245.2, 203.2, 174.1, 131.1, 103.1, 77.1. ${\left[\alpha \right]}_{25}^{D}$ = +69.0 (*c*, 0.05, CH_2_Cl_2_). ^1^H/^13^C NMR (400/100 MHz, CDCl_3_), see Table 1.

Bufanidrine (**2**), GC-MS: R_t_ 23.138 min; MS; *m*/*z*: 315.3 (C_18_H_21_NO_4_), 300.3, 260.2, 245.2, 231.2, 202.2, 115.1. ${\left[\alpha \right]}_{25}^{D}$ = −138.1 (*c*, 0.04, CH_2_Cl_2_). ^1^H/^13^C NMR (400/100 MHz, CDCl_3_), see Table 1.

Buphanisine (**3**), GC-MS: R_t_ 20.350 min; MS; *m*/*z*: 285.3 (C_17_H_19_NO_3_), 254.2, 215.2, 157.2, 115.1, 77.1. ${\left[\alpha \right]}_{25}^{D}$ = −78.8 (*c*, 0.04, CH_2_Cl_2_). ^1^H/^13^C-NMR (400/100 MHz, CDCl_3_), see Table 1.

Epibuphanisine (**4**), GC-MS: R_t_ 20.285 min; MS; *m*/*z*: 285.3 (C_17_H_19_NO_3_), 254.2, 215.2, 157.2, 115.1, 77.1. ${\left[\alpha \right]}_{25}^{D}$ = +8.8 (*c*, 0.2, CH_2_Cl_2_). ^1^H/^13^C NMR (400/100 MHz, CDCl_3_), see Table 1.

### 4.4. Cell Culture and Maintenance

The human neuroblastoma SH-SY5Y cells were generously donated by the Blackburn Laboratory, University of Cape Town. Cells were grown in Dulbecco’s modified Eagle’s Mmedium (DMEM, Gibco, Life Technologies Corporation, Paisley, UK), supplemented with 10% fetal bovine serum, (FBS, Gibco, Life Technologies Corporation, Paisley, UK), 100 U/mL penicillin, and 100 µg/mL streptomycin (Lonza Group Ltd., Verviers, Belgium). Cultures were incubated at 37 °C in humidified air with 5% CO_2_ with a medium change every three days. Cells were sub-cultured when they attained 70 to 80 percent confluency using a solution of 0.25% trypsin EDTA (Lonza Group Ltd., Verviers, Belgium).

### 4.5. Treatments

Stock solutions of 40 mg/mL of *C. flava* extract as well as compounds were prepared in dimethyl sulfoxide (DMSO) (Sigma–Aldrich, St. Louis, MO, USA) from which final concentrations were made in cell growth medium. To determine the optimum concentration of *C. flava* and compounds to be used for neuroprotection studies, SH-SY5Y cells were plated at a density of 10,000 cells/well and treated with concentrations (2.5, 5, and 10 µg/mL) of the extract of *C. flava* and compounds (**1**, **2**, **3**, and **4**) (Table 1). The vehicle-treated cells (cells treated with the same concentration of DMSO like that of the highest concentration of extract or compounds) were used as the control. All treatments lasted for 24 h, and the 2.5 µg/mL concentration was selected for the neuroprotection studies. Cells were plated as described above and pre-treated with 2.5 µg/mL of the *C. flava* extract and compounds for 2 h prior to the addition of 2000 µM MPP^+^ to the extract, and compounds containing wells and treatments were incubated for 24 h [64,65]. The concentration of MPP^+^ used for this study was informed by a previous study from our laboratory [45] and the untreated cells served as the control.

### 4.6. Cell Viability Assays

The MTT (Sigma–Aldrich, St. Louis, MO, USA) cell viability assay was used to determine the viability of cells following treatment with both plant extracts and MPP^+^. Cells were seeded in 96-well plates and treated as stated above after which the MTT assay was performed. Briefly, after treatment, 10 or 20 µL (depending on well volume) of 5 mg/mL MTT solution in PBS (Lonza Group Ltd., Verviers, Belgium) was added to each well and left to incubate in the dark at 37 °C for 4 h. After incubation, the medium containing the MTT dye was discarded, and the MTT formazan was solubilized with 100 μL of DMSO for absorbance reading using a microplate reader (BMG Labtech Omega^®^ POLARStar) at a wavelength of 570 nm. Cell viability was calculated and expressed as percentage of control.

### 4.7. Cell Morphology

To visualize changes in cell morphology of the SH-SY5Y cells following the respective treatments, cells were seeded in 96-well plates at a density of 10,000 cells per well and pre-treated with 2.5 µg/mL of *C. flava* extract and compounds for 2 h prior to the addition of 2000 µM of MPP^+^. After the 24 h treatment, changes in morphology for the various treatment conditions were observed using the Zeiss inverted light microscope with 10× objective lens. Images were captured using the Zeiss software version 2.3.

### 4.8. Reactive Oxygen Species (ROS) Assay

To ascertain whether *C. flava* and compounds impacted on MPP^+^-induced ROS production, SH-SY5Y cells were plated in black 96-well plates and following overnight attachment, the cells were exposed to *C. flava* and compounds before introduction of MPP^+^. At the termination of the experiment after 24 h, cells were washed with PBS and stained with 20 µM of 2′,7′-dichlorofluorescin diacetate (DCFDA, Sigma–Aldrich, St. Louis, MO, USA) fluorescent dye diluted in un-supplemented DMEM for 1 h. Thereafter, the dye-containing medium was aspirated, and cells were washed again with PBS and the fluorescence intensity of the DCFDA dye in the cells was read in PBS using a microplate reader (BMG Labtech Omega^®^ POLARStar), and values obtained were expressed as percentages of control.

### 4.9. Adenosine Triphosphate Assay

The Mitochondrial ToxGlo ATP assay kit (Promega, Madison, WI, USA) was used to investigate ATP levels in the cells. Briefly, cells were plated at a density of 10,000 cells per well in a white 96-well plate and after attachment, cells were treated as per neuroprotection assay above. After treatment, cells were processed according to the manufacturer’s protocol, the luminescence intensity was read using the microplate reader (BMG Labtech Omega^®^ POLARStar), and readings were expressed as percentages of the control.

### 4.10. Caspase 3/7 Apoptosis Assay

To investigate apoptosis in the cells, the caspase 3/7 assay kit (Promega, Madison, WI, USA) was used to estimate levels of apoptosis in the cells according to the manufacturer’s instructions. Briefly, cells were plated in a white 96-well plate at a density of 10,000 cells per well and allowed to attach overnight, after which cells were pre-treated with *C. flava* and compounds before the addition of 2000 µM MPP^+^. Treatments lasted for 24 h and at the end of the experiments, equal volumes of the caspase 3/7 assay mix were added to each well, and the luminescence intensity was read with a microplate reader (BMG Labtech Omega^®^ POLARStar). The luminescence intensity of treated cells was expressed as percentages of the control.

### 4.11. Statistical Analysis

Data generated from this study were expressed as means ± standard error of means of at least three independent experiments analyzed using GraphPad Prism version 6. Significance between groups was determined using one-way analysis of variance (ANOVA). A value of *p* < 0.05 was considered significant.

## 5. Conclusions

This study investigated the neuroprotective effects of *C. flava* and its isolated bioactive compounds in an in vitro model of PD. Four alkaloids were isolated, and we presented the first evidence of the activities of these compounds and the *C. flava* extract in a PD model. As a mechanism of action, it was found that both the extract and the compounds attenuated ATP levels in the cells and inhibited MPP^+^-induced apoptosis. This further lends credence to the acclaimed traditional use of the Amaryllidaceae family to treat nervous system disorders as well as shows the potential of exploiting Amaryllidaceae alkaloids for novel drug candidates.

## Figures and Tables

**Table 1:** ^1^H (400 MHz) and ^13^C (100 MHz) NMR data of compounds **1**–**4** in CDCl_3_.

	Pancratinine B (1)		Bufanidrine (2)		Buphanisine (3)		Epibuphanisine (4)	
No	δ_C_	δ, *J* (Hz)	δ_C_	δ, *J* (Hz)	δ_C_	δ, *J* (Hz)	δ_C_	δ, *J* (Hz)
1	133.4 *d*	6.03 *br d*, 10.4	132.9 *d*	6.58 *d*, 10.2	132.9 *d*,	6.55 *d*, 9.8	132.1 *d*	6.58 *d*, 10.4
2	131.7 *d*	5.70 *br d*, 10.4	125.3 *d*	5.95 *dd*, 10.2, 5.2	125.4 *d*,	5.95 *dd*, 9.8, 5.2	125.6 *d*	5.96 *dd*, 10.4, 5.4
3	72.3 *d*	3.94 *br dd*, 5.0, 10.8	72.6 *d*	3.82 *	77.2 *d*	3.81 *	72.3 *d*	3.80 *ddd*, 5.4, 4.3, 1.7
4	30.9 *t*	1.50 *ddd*, 4.6, 11.0, 12.8	28.7 *t*	2.08 *m*	28.7 *t*,	2.07 *ddd*, 13.3, 4.3, 2.0	28.2 *t*	2.20 *
		2.35 *br d*, 12.9		1.59 *dt*, 13.7, 4.0		1.58 *td*, 13.3, 4.3		1.62 *td*, 13.4, 4.2
4a	67.5 *d*	2.95 *m*	62.6 *d*	3.31 *dd*, 13.7, 4.0	63.1 *d*	3.43 ^#^	63.3 *d*	3.32 **
6	61.8 *t*	3.83 *d*, 16.8	58.6 *t*	4.24 *d*, 17.6	62.3 *t*	4.45 *d*, 16.7	61.8 *t*	4.38 *d*, 16.7
		4.26 *d*, 16.8		3.81 * *d*, 17.6		3.83 * *d*, 16.7		3.75 *d*, 16.7
6a	125.4 *s*		117.3 *s*		126.2 *s*		125.6 *s*	
7	106.8 *d*	6.51 *s*	139.4 *s*		106.9 *s*	6.45 *s*	106.9 *d*	6.46 *s*
8	145.9 *s*		133.4 *s*		145.7 *s*		145.9 *s*	
9	147.4 *s*		148.0 *s*		146.0 *s*		146.4 *s*	
10	109.9 *d*	6.63 *s*	96.6 *d*	6.81 *s*	102.9 *d*	6.82 *s*	103.0 *d*	6.81 *s*
10a	129.4 *s*		139.3 *s*		138.4 *s*		137.7 *s*	
10b			44.3 *s*		44.3 *s*		44.5 *s*	
11 (*exo*)	49.2 *t*	2.64 *d*, 2.2	44.0 *t*	2.15, *ddd*, 12.7, 9.8, 4.8	44.2 *t*	2.15 *ddd*, 12.4, 9.2, 4.3	43.6 *t*	1.89 *ddd*, 10.6, 10.6, 5.2
11 (*endo*)				1.90 *ddd*, 11.8, 10.6, 5.8		1.90 *ddd*, 12.4, 11.0, 6.0		2.20 *
11b	82.5 *s*							
12 (*exo*)	54.7 *t*	2.82 *d*, 11.8	53.6 *t*	3.37 **	53.4 *t*	3.45 ^#^	53.3 *t*	3.46
12 (*endo*)		2.97 *dd*, 2.2, 11.8		2.87, *ddd*, 14.0, 9.0, 6.0		2.93 *ddd*, 12.0, 9.0, 6.0		2.94 *ddd*, 14.0, 9.0, 6.0
OCH_2_O	100.9 *t*	5.92 *s*	100.5 *t*	5.85, 5.83 *d*/each, 1.40	100.7 *t*	5.88, 5.87 *br s*/each	100.9 t	5.86, 5.85 *d*, 1.3
OMe-3	56.3 *q*	3.42 *s*	56.4 *q*	3.35 ** *s*	56.5 *q*	3.34 *s*	56.6 *q*	3.34 ** *s*
OMe-7			59.1 *q*	3.96 *s*				

*, ** Overlapped signals in the same column.

## Data Availability

Not Applicable.

## Sample Availability

Samples of the compounds **1**, **2**, **3**, and **4** are available from the authors on request.

## References

[1] Ferreira S.A., Romero-Ramos M. (2018). Microglia response during Parkinson’s disease: Alpha-synuclein intervention. Front. Cell. Neurosci..

[2] Massano J., Bhatia K.P. (2012). Clinical approach to Parkinson’s disease: Features, diagnosis, and principles of management. Cold Spring Harb. Perspect. Med..

[3] Kaidery N.A., Thomas B. (2018). Current perspective of mitochondrial biology in Parkinson’s disease. Neurochem. Int..

[4] Ma J., Gao J., Wang J., Xie A. (2019). Prion-like mechanisms in Parkinson’s disease. Front. Neurosci..

[5] Maiti P., Manna J., Dunbar G.L. (2017). Current understanding of the molecular mechanisms in Parkinson’s disease: Targets for potential treatments. Transl. Neurodegener..

[6] Dias V., Junn E., Mouradian M.M. (2013). The role of oxidative stress in Parkinson’s disease. J. Parkinson’s Dis..

[7] Kieburtz K., Wunderle K.B. (2013). Parkinson’s disease: Evidence for environmental risk factors. Mov. Disord. Off. J. Mov. Disord. Soc..

[8] Chen H., Ritz B. (2018). The search for environmental causes of Parkinson’s disease: Moving forward. J. Parkinson’s Dis..

[9] Lee T.K., Yankee E.L. (2021). A review on Parkinson’s disease treatment. Neuroimmunol. Neuroinflamm..

[10] Zahoor I.S.A., Haq E., Stoker T.B., Greenland J.C. (2018). Pharmacological Treatment of Parkinson’s Disease. Parkinson’s Disease: Pathogenesis and Clinical Aspects.

[11] Surguchov A., Bernal L., Surguchev A.A. (2021). Phytochemicals as Regulators of Genes Involved in Synucleinopathies. Biomolecules.

[12] Rabiei Z., Solati K., Amini-Khoei H. (2019). Phytotherapy in treatment of Parkinson’s disease: A review. Pharm. Biol..

[13] Ekor M. (2014). The growing use of herbal medicines: Issues relating to adverse reactions and challenges in monitoring safety. Front. Pharmacol..

[14] Li Q., Zhao D., Bezard E. (2006). Traditional Chinese medicine for Parkinson’s disease: A review of Chinese literature. Behav. Pharmacol..

[15] Freitas M.E., Hess C.W., Fox S.H. (2017). Motor Complications of Dopaminergic Medications in Parkinson’s Disease. Semin. Neurol..

[16] Fennell C.W., van Staden J. (2001). *Crinum* species in traditional and modern medicine. J. Ethnopharmacol..

[17] Koekemoer M., Steyn H.M., Bester S.P. (2013). Guide to Plant Families of Southern Africa.

[18] Van Goietsenoven G., Andolfi A., Lallemand B., Cimmino A., Lamoral-Theys D., Gras T., Abou-Donia A., Dubois J., Lefranc F., Mathieu V. (2010). Amaryllidaceae Alkaloids Belonging to Different Structural Subgroups Display Activity against Apoptosis-Resistant Cancer Cells. J. Nat. Prod..

[19] He M., Qu C., Gao O., Hu X., Hong X. (2015). Biological and pharmacological activities of Amaryllidaceae alkaloids. RSC Adv..

[20] Naidoo D., Roy A., Slavětínská L.P., Chukwujekwu J.C., Gupta S., Van Staden J. (2020). New role for crinamine as a potent, safe and selective inhibitor of human monoamine oxidase B: In vitro and in silico pharmacology and modeling. J. Ethnopharmacol..

[21] Sibanyoni M.N., Chaudhary S.K., Chen W., Adhami H.-R., Combrinck S., Maharaj V., Schuster D., Viljoen A. (2020). Isolation, in vitro evaluation and molecular docking of acetylcholinesterase inhibitors from South African Amaryllidaceae. Fitoterapia.

[22] Heinrich M., Teoh H.L. (2004). Galanthamine from snowdrop—the development of a modern drug against Alzheimer’s disease from local Caucasian knowledge. J. Ethnopharmacol..

[23] Viladomat F., Bastida J., Codina C., Campbell W.E., Mathee S. (1995). Alkaloids from *Boophane flava*. Phytochemistry.

[24] Viladomat F., Codina C., Bastida J., Mathee S., Campbell W.E. (1995). Further alkaloids from *Brunsvigia josephinae*. Phytochemistry.

[25] Berkov S., Osorio E., Viladomat F., Bastida J. (2020). Chemodiversity, chemotaxonomy and chemoecology of Amaryllidaceae alkaloids. The Alkaloids: Chemistry and Biology.

[26] Cedron J.C., Oberti J.C., Estevez-Braun A., Ravelo A.G., Del Arco-Aguilar M., Lopez M. (2009). *Pancratium canariense* as an important source of Amaryllidaceae alkaloids. J. Nat. Prod..

[27] Liu W.-B., Zhou J., Qu Y., Li X., Lu C.-T., Xie K.-L., Sun X.-L., Fei Z. (2010). Neuroprotective effect of osthole on MPP^+^-induced cytotoxicity in PC12 cells via inhibition of mitochondrial dysfunction and ROS production. Neurochem. Int..

[28] Abushouk A.I., Negida A., Ahmed H., Abdel-Daim M.M. (2017). Neuroprotective mechanisms of plant extracts against MPTP induced neurotoxicity: Future applications in Parkinson’s disease. Biomed. Pharmacother..

[29] Yan M.H., Wang X., Zhu X. (2013). Mitochondrial defects and oxidative stress in Alzheimer disease and Parkinson disease. Free Radic. Biol. Med..

[30] Requejo-Aguilar R., Bolaños J.P. (2016). Mitochondrial control of cell bioenergetics in Parkinson’s disease. Free Radic. Biol. Med..

[31] Sehgal P., Szalai P., Olesen C., Praetorius H.A., Nissen P., Christensen S.B., Engedal N., Møller J.V. (2017). Inhibition of the sarco/endoplasmic reticulum (ER) Ca^2+^-ATPase by thapsigargin analogs induces cell death via ER Ca^2+^ depletion and the unfolded protein response. J. Biol. Chem..

[32] Nakajima Y.-i., Kuranaga E. (2017). Caspase-dependent non-apoptotic processes in development. Cell Death Differ..

[33] Li J., Yuan J. (2008). Caspases in apoptosis and beyond. Oncogene.

[34] Thornberry N.A., Rano T.A., Peterson E.P., Rasper D.M., Timkey T., Garcia-Calvo M., Houtzager V.M., Nordstrom P.A., Roy S., Vaillancourt J.P. (1997). A combinatorial approach defines specificities of members of the caspase family and granzyme B Functional relationships established for key mediators of apoptosis. J. Biol. Chem..

[35] Bressenot A., Marchal S., Bezdetnaya L., Garrier J., Guillemin F., Plénat F. (2009). Assessment of apoptosis by immunohistochemistry to active caspase-3, active caspase-7, or cleaved PARP in monolayer cells and spheroid and subcutaneous xenografts of human carcinoma. J. Histochem. Cytochem..

[36] Obeso J., Stamelou M., Goetz C., Poewe W., Lang A., Weintraub D., Burn D., Halliday G.M., Bezard E., Przedborski S. (2017). Past, present, and future of Parkinson’s disease: A special essay on the 200th Anniversary of the Shaking Palsy. Mov. Disord..

[37] McDonald C., Gordon G., Hand A., Walker R.W., Fisher J.M. (2018). 200 Years of Parkinson’s disease: What have we learnt from James Parkinson?. Age Ageing.

[38] Voon V., Napier T.C., Frank M.J., Sgambato-Faure V., Grace A.A., Rodriguez-Oroz M., Obeso J., Bezard E., Fernagut P.-O. (2017). Impulse control disorders and levodopa-induced dyskinesias in Parkinson’s disease: An update. Lancet Neurol..

[39] Espay A.J., Morgante F., Merola A., Fasano A., Marsili L., Fox S.H., Bezard E., Picconi B., Calabresi P., Lang A.E. (2018). Levodopa-induced dyskinesia in Parkinson disease: Current and evolving concepts. Ann. Neurol..

[40] Lee D.J., Dallapiazza R.F., De Vloo P., Lozano A.M. (2018). Current surgical treatments for Parkinson’s disease and potential therapeutic targets. Neural Regen. Res..

[41] Nair J.J., van Staden J. (2014). Cytotoxicity studies of lycorine alkaloids of the Amaryllidaceae. Nat. Prod. Commun..

[42] Adewusi E.A., Fouche G., Steenkamp V. (2012). Cytotoxicity and acetylcholinesterase inhibitory activity of an isolated crinine alkaloid from *Boophane disticha* (Amaryllidaceae). J. Ethnopharmacol..

[43] Nair J.J., van Staden J. (2013). Pharmacological and toxicological insights to the South African Amaryllidaceae. Food Chem. Toxicol..

[44] Jin A., Li X., Zhu Y.-Y., Yu H.-Y., Pi H.-F., Zhang P., Ruan H.-L. (2014). Four new compounds from the bulbs of *Lycoris aurea* with neuroprotective effects against CoCl_2_ and H_2_O_2_-induced SH-SY5Y cell injuries. Arch. Pharmacal Res..

[45] Omoruyi S.I., Delport J., Kangwa T.S., Ibrakaw A.S., Cupido C.N., Ekpo O.E., Hussein A.A. (2020). In vitro neuroprotective potential of *Clivia miniata* and *Nerine humilis* (Amaryllidaceae) in MPP^+^-induced neuronal toxicity in SH-SY5Y neuroblastoma cells. S. Afr. J. Bot..

[46] Cimmino A., Masi M., Evidente M., Superchi S., Evidente A. (2017). Amaryllidaceae alkaloids: Absolute configuration and biological activity. Chirality.

[47] Ding Y., Qu D., Zhang K.-M., Cang X.-X., Kou Z.-N., Xiao W., Zhu J.-B. (2017). Phytochemical and biological investigations of Amaryllidaceae alkaloids: A review. J. Asian Nat. Prod. Res..

[48] Hulcová D., Breiterová K., Siatka T., Klímová K., Davani L., Šafratová M., Hošťálková A., De Simone A., Andrisano V., Cahlíková L. (2018). Amaryllidaceae alkaloids as potential glycogen synthase kinase-3β inhibitors. Molecules.

[49] Sandager M., Nielsen N.D., Stafford G.I., van Staden J., Jäger A.K. (2005). Alkaloids from *Boophane disticha* with affinity to the serotonin transporter in rat brain. J. Ethnopharmacol..

[50] Elgorashi E.E., Stafford G.I., Jäger A.K., van Staden J. (2006). Inhibition of [3H] citalopram binding to the rat brain serotonin transporter by Amaryllidaceae alkaloids. Planta Med..

[51] Neergaard J.S., Andersen J., Pedersen M.E., Stafford G.I., Staden J.V., Jäger A.K. (2009). Alkaloids from *Boophone disticha* with affinity to the serotonin transporter. S. Afr. J. Bot..

[52] Crews L., Masliah E. (2010). Molecular mechanisms of neurodegeneration in Alzheimer’s disease. Hum. Mol. Genet..

[53] Huang W.-J., Zhang X., Chen W.-W. (2016). Role of oxidative stress in Alzheimer’s disease. Biomed. Rep..

[54] Elgorashi E.E., Stafford G.I., Van Staden J. (2004). Acetylcholinesterase enzyme inhibitory effects of Amaryllidaceae alkaloids. Planta Med..

[55] Moon H.E., Paek S.H. (2015). Mitochondrial Dysfunction in Parkinson’s Disease. Exp. Neurobiol..

[56] Burté F., De Girolamo L.A., Hargreaves A.J., Billett E.E. (2011). Alterations in the mitochondrial proteome of neuroblastoma cells in response to complex 1 inhibition. J. Proteome Res..

[57] Piao Y., Kim H.G., Oh M.S., Pak Y.K. (2012). Overexpression of TFAM, NRF-1 and myr-AKT protects the MPP^+^-induced mitochondrial dysfunctions in neuronal cells. Biochim. Biophys. Acta (BBA) Gen. Subj..

[58] Jeong K.H., Jeon M.-T., Kim H.D., Jung U.J., Jang M.C., Chu J.W., Yang S.J., Choi I.Y., Choi M.-S., Kim S.R. (2015). Nobiletin protects dopaminergic neurons in the 1-methyl-4-phenylpyridinium-treated rat model of Parkinson’s disease. J. Med. Food.

[59] Zhang X., Zhou J.-Y., Chin M.H., Schepmoes A.A., Petyuk V.A., Weitz K.K., Petritis B.O., Monroe M.E., Camp D.G., Wood S.A. (2010). Region-specific protein abundance changes in the brain of MPTP-induced Parkinson’s disease mouse model. J. Proteome Res..

[60] Lepule K.H., Cordier W., Steenkamp P., Nell M., Steenkamp V. (2019). The ability of three African herbal remedies to offer protection against an in vitro model of Parkinson’s disease. S. Afr. J. Bot..

[61] Ito K., Eguchi Y., Imagawa Y., Akai S., Mochizuki H., Tsujimoto Y. (2017). MPP^+^ induces necrostatin-1- and ferrostatin-1-sensitive necrotic death of neuronal SH-SY5Y cells. Cell Death Discov..

[62] Julien O., Wells J.A. (2017). Caspases and their substrates. Cell Death Differ..

[63] Waldmeier P.C., Tatton W.G. (2004). Interrupting apoptosis in neurodegenerative disease: Potential for effective therapy?. Drug Discov. Today.

[64] Egunlusi A.O., Malan S.F., Omoruyi S.I., Ekpo O.E., Palchykov V.A., Joubert J. (2020). Open and rearranged norbornane derived polycyclic cage molecules as potential neuroprotective agents through attenuation of MPP^+^-and Calcium overload-induced excitotoxicity in neuroblastoma SH-SY5Y cells. Eur. J. Med. Chem..

[65] Ibrakaw A.S., Omoruyi S.I., Ekpo O.E., Hussein A.A. (2020). Neuroprotective activities of *Boophone haemanthoides* (Amaryllidaceae) extract and its chemical constituents. Molecules.

